# Freeze–thaw performance of self-compacting concrete with GGBFS, fly ash and limestone powder: linking strength loss to air-void parameters

**DOI:** 10.1038/s41598-026-52477-9

**Published:** 2026-05-09

**Authors:** Tomasz Rudnicki, Piotr Smarzewski

**Affiliations:** Faculty of Civil Engineering and Geodesy, Military University of Technology, 2 Gen. Sylwestra Kaliskiego, 00-908 Warsaw, Poland

**Keywords:** Self-compacting concrete (SCC), Supplementary cementitious materials (SCMs), GGBFS, Fly ash, Limestone powder, Freeze–thaw resistance, Air-void spacing factor (L), Microstructure (SEM/EDS), Engineering, Materials science

## Abstract

Comparative evidence on how different supplementary cementitious materials affect freeze–thaw performance and air-void system characteristics of self-compacting concrete (SCC) under consistent mixture conditions remains limited. This study provides a direct comparative assessment of ground granulated blast-furnace slag (GGBFS), fly ash (FA), and limestone powder (LM) as partial cement replacements in SCC within a unified mix-design framework. SCC mixtures incorporating 15% and 30% of GGBFS, FA, or LM were evaluated for fresh properties (slump flow, V-funnel, L-box) and for mechanical performance (compressive and splitting tensile strength, modulus of elasticity) at 28, 56, and 90 days. Freeze–thaw performance was evaluated after 150 cycles, while the hardened air-void system was characterized using total air content (A), micro-air content (A_300_), and spacing factor (L). Selected mixtures were examined by SEM/EDS and complemented by a limited quantitative SEM texture assessment at fixed magnification to support microstructural interpretation. Statistical analysis (two-way ANOVA and Tukey HSD) and regression-based relationships were used to strengthen the comparison. The results show that FA primarily improved flowability, reaching a slump flow of 740 mm at 30% replacement, whereas GGBFS provided the most favorable balance between late-age mechanical performance and freeze–thaw resistance, achieving 69 MPa compressive strength at 90 days and only 0.2% mass loss and 1.0% strength loss after F150 at 30% replacement. LM contributed to stabilizing the protective air-void network, while the lowest spacing factor (L = 0.11 mm) was observed for GGBFS30 and LM30. Importantly, freeze–thaw strength loss was more strongly associated with spacing factor L than with total air content, highlighting void spacing as a practical durability-oriented predictor for SCC incorporating SCMs. Overall, the findings support clinker reduction strategies while maintaining durable SCC performance.

## Introduction

Self-compacting concrete (SCC) is widely used because it can flow and consolidate under its own weight without vibration, enabling efficient placement and high-quality finishes, particularly in heavily reinforced or complex structural elements^1–6^.

Mineral admixtures strongly influence both the fresh-state behavior and hardened performance of SCC. Among the most commonly used are ground granulated blast furnace slag (GGBFS), fly ash (FA), and limestone powder (LM). These materials can improve rheology through particle packing and paste modification, while reactive SCMs may also contribute to later-age strength development and microstructural refinement^7–13^.

In parallel, increasing attention has been paid to optimizing granular composition and particle packing in SCC. Improved packing not only enhances matrix density but also enables a reduction in Portland cement content, supporting significant reductions in CO_2_ emissions^14,15^. Within this context, GGBFS has been consistently associated with improved late-age strength development and refined microstructure due to its reactivity and contribution to secondary hydrate formation^16,17^. Fly ash (particularly Class F) can enhance deformability and durability-related performance, benefiting from its spherical particle morphology and chemical composition, albeit often with delayed early-age strength contribution compared with more hydraulically reactive binders^16–18^. Limestone powder, although largely inert, is widely used as a fine filler that densifies the binder matrix, improves homogeneity, and mitigates microstructural discontinuities through packing and nucleation effects^18–20^.

Beyond performance, partial replacement of Portland cement with supplementary cementitious materials (SCMs) contributes to broader sustainability goals. These mineral additions are often industrial by-products, aligning with circular economy principles by reducing waste and lowering demand for energy-intensive clinker production. For example, fluidized bed combustion fly ash has been reported as a feasible partial cement replacement in underwater concrete with satisfactory performance and environmental benefit^21^, while silica-rich fly ash contributes to sustainable waste management strategies^22^. At a system level, recent reviews emphasize that broad SCM implementation is essential to decarbonize cementitious supply chains in Europe, particularly under climate neutrality targets^23^.

Recent studies on hybrid SCM systems and sustainable cementitious mixtures indicate that the interaction between binder chemistry, microstructural development, and performance is often multi-mechanistic rather than linear. In particular, blended systems incorporating industrial by-products and fillers may modify rheology, strength development, pore structure, and durability-related response through coupled packing, hydration, and microstructural refinement effects. This has been demonstrated both for self-compacting cementitious systems containing blended SCMs and for sustainable SCC mixtures incorporating slag-based components, where combined material substitutions influenced fresh-state behavior, mechanical performance, and durability in a non-uniform manner^24,25^.

The relevance of this research is also rooted in the growing demand for durable and sustainable concrete solutions. SCC, due to its excellent flowability, homogeneity, and ability to fill complex formwork and congested reinforcement zones, provides a practical response to modern construction constraints^26–28^. As durability and sustainability increasingly influence engineering design, SCM-based SCC mixtures are being adopted to reduce clinker content while aiming to maintain long-term performance.

A large body of literature confirms the beneficial effects of SCMs on SCC behavior. For instance, GGBFS has been reported to improve durability and reduce permeability of the concrete matrix^29^, while FA is commonly associated with improved flowability and enhanced later-age mechanical performance^30,31^. Limestone powder has been shown to improve viscosity and volumetric stability, contributing to robust self-compacting behavior when appropriately proportioned^32–40^.

Previous studies have shown that the freeze–thaw durability of SCC can vary substantially depending on the type of supplementary materials and the adopted test procedure. For example, in SCC incorporating fly ash and tuff powder, the mass loss after 250 freeze–thaw cycles decreased from 3.7% in the reference mixture to 2.1% in the mixture containing 20% fly ash, while the corresponding reduction in relative dynamic elastic modulus decreased from 18.3% to 7%^41^. In another study on SCC containing mineral admixtures and nano-clay, the mass loss after 300 cycles decreased from 18.6% in the control mixture to 4% in the mixture modified with 3% nano-clay, and the corresponding reduction in dynamic modulus decreased from 44.9% to 15.1%^42^. These results indicate that compositional modifications may significantly improve SCC frost resistance. However, direct comparison between studies remains difficult because the mixtures, curing regimes, freeze–thaw procedures, and durability indicators are not uniform. This highlights the need for internally consistent comparative studies that relate freeze–thaw deterioration to hardened air-void system characteristics under identical SCC mixture-design conditions. Recent work has further emphasized that freeze-related damage should be interpreted not only through strength loss, but also through pore-structure evolution and microstructural indicators, since improved freeze resistance has been associated with reduced macropore content and a denser cementitious matrix^43^.

Despite extensive research on mineral additions in SCC, important gaps remain. Existing studies commonly examine single SCM systems, selected fresh or mechanical properties, or freeze–thaw indicators in isolation, and direct comparison between published results is further limited by differences in mixture design, curing regime, test procedure, and evaluation criteria. As a result, directly comparable datasets that systematically assess GGBFS, FA, and LM within one unified SCC framework and relate freeze–thaw deterioration to hardened air-void system characteristics remain scarce. Consequently, it is still insufficiently resolved to what extent frost durability is governed by total air content, void-spacing efficiency, SCM-induced matrix refinement, or the combined action of these factors.

In this context, the present study aims to comprehensively assess the influence of three commonly used mineral admixtures (GGBFS, FA, and LM) on key performance indicators of SCC, including workability, compressive strength, freeze–thaw resistance, air-void system parameters, and modulus of elasticity. Given the time-dependent behavior of these materials, performance is evaluated at three curing ages: 28, 56, and 90 days. Particular emphasis is placed on interpreting freeze–thaw performance in relation to hardened air-void system characteristics within one internally consistent comparative framework.

The novelty of this study lies in three main aspects. First, it provides a direct comparative assessment of GGBFS, FA, and LM in SCC under a unified mix-design and consistent experimental framework. Second, freeze–thaw resistance is interpreted through hardened air-void system characteristics (A, A_300_, and L), enabling durability deterioration to be related directly to the efficiency of the protective void network under identical SCC conditions. Third, this interpretation is supported by selected SEM/EDS observations and a limited quantitative SEM texture assessment at fixed magnification, providing complementary microstructural context for the comparative results.

## Materials and methods

### Materials

The self-compacting concrete (SCC) mixtures were produced using Portland cement CEM I 42.5 R as the primary binder, with ground granulated blast-furnace slag (GGBFS), Class F fly ash (FA), and limestone powder (LM) applied as partial cement replacements. Natural aggregates (maximum size 16 mm) and potable tap water were used throughout.

Portland cement CEM I 42.5 R was supplied by the Odra cement plant (Opole, Poland) and complied with PN-EN 197-1^44^. The cement had a Blaine fineness of 4260 cm^2^/g and a specific gravity of 3.11 g/cm^3^. As summarized in Table 1, the cement exhibited high early strength (30.6 MPa after 2 days and 51.3 MPa after 28 days), good volumetric stability (Le Chatelier expansion 0.9 mm), and an initial setting time of 203 min, meeting the relevant standard requirements^45^.

The GGBFS was obtained from a local supplier and conformed to PN-EN 15167-1^45^. Its specific gravity was 2.90 g/cm^3^ and Blaine fineness was approximately 4500 cm^2^/g (Table 1). GGBFS is commonly associated with pronounced later-age strength development, particularly beyond 56–90 days, due to continued reactions and progressive microstructural refinement^38–40^.

Fly ash was a low-calcium Class F material derived from hard coal combustion and meeting ASTM C618 requirements^46^. The FA had a specific gravity of 2.40 g/cm^3^ (Table 1). Owing to its particle morphology and composition, FA typically enhances flowability and workability of fresh concrete; while it may delay early-age strength development, it contributes to long-term strength and durability^16,17^.

The limestone powder was ground to a Blaine fineness of 2800 cm^2^/g and had a specific gravity of 2.70 g/cm^3^ (Table 1). Although LM is largely inert, it acts as a fine filler that improves particle packing and matrix homogeneity, supporting densification and reduced porosity^19,20^.

The key properties of the mineral admixtures and cement are listed in Table 1.

**Table 1:** Physical properties and chemical composition of cementitious materials used in SCC mixtures.

Material	Standard	Physical properties		Chemical composition (%)						Other main constituents/remarks
		Specific gravity (g/cm^3^)	Blaine fineness (cm^2^/g)	CaO	SiO_2_	Al_2_O_3_	Fe_2_O_3_	MgO	SO₃	
CEM I 42.5 R	PN-EN 197-1^44^	3.11	4260	64.6	20.2	4.3	3.3	–	2.5	Cl⁻: 0.07%; compressive strength: 30.6 MPa (2 d), 51.3 MPa (28 d); initial setting time: 203 min
GGBFS	PN-EN 15167-1^45^	2.90	4500	40.0	35.0	10.0	3.0	8.0	–	–
FA	ASTM C618^46^	2.40	–	5.0	55.0	25.0	8.0	2.0	–	Class F fly ash
LM	–	2.70	2800	–	2.0	0.5	–	–	–	CaCO_3_: 94%; MgCO_3_: 3%

Natural river sand (maximum size 2 mm) was used as the fine aggregate (specific gravity 2.65 g/cm^3^; bulk density 1.50 kg/dm^3^). Coarse aggregate consisted of gravel fractions 2–8 mm and 8–16 mm (specific gravity 2.65 g/cm^3^; bulk density 1.45 kg/dm^3^).

Mixing water complied with PN-EN 1008^47^. Two chemical admixtures were used: a polycarboxylate ether (PCE) superplasticizer and an air-entraining admixture compatible with CEM I 42.5 R. In the final SCC mixtures, their dosages were kept constant for all compositions, as given in Table 2 (8 kg/m^3^ of PCE superplasticizer and 2.4 kg/m^3^ of air-entraining admixture).

### Mix design

The SCC mix design procedure followed the methodology described in^6,13,18^ and the recommendations of Khayat and de Schutter^12^. The binder content (cement plus mineral admixture) was fixed at 400 kg/m^3^, and the water-to-binder ratio (w/b) was maintained at 0.40. A total binder content of 400 kg/m^3^ was adopted as a representative SCC powder level that allows adequate paste volume, flowability, and mixture stability without excessive powder demand, while remaining consistent with commonly reported SCC proportioning approaches^6,12,13,18^. The fine-to-coarse aggregate ratio was kept constant at 40:60 by mass. Within this framework, GGBFS, FA, or LM was introduced as a partial cement replacement by mass at two levels, 15% and 30%, while the reference mixture contained no mineral addition. These replacement levels were chosen to represent moderate and relatively high substitution ranges commonly reported in the literature for SCC incorporating slag, fly ash, or limestone powder, while maintaining workable and technically realistic mixtures for direct comparison^16–20^. For each SCM, a separate series of mixtures was prepared, including a reference mix and two replacement levels (15% and 30%).

The full compositions are given in Table 2, which reports the exact contents of cement, mineral admixtures, water, aggregates, and chemical admixtures for all SCC mixtures. The water content was kept constant throughout, with no correction for SCM type or reactivity, so that the effect of binder substitution could be evaluated under the same nominal w/b ratio of 0.40.

**Table 2:** Mix proportions of the investigated self-compacting concrete (SCC) mixtures (kg/m^3^).

Mix ID	Cement	GGBFS	FA	LM	Water	Fine aggregate 0–2 mm	Coarse aggregate 2–8 mm	Coarse aggregate 8–16 mm	PCE superplasticizer	Air-entraining admixture
REF	400	0	0	0	160	742	427	686	8	2.4
GGBFS15	340	60	0	0	160	740	426	684	8	2.4
GGBFS30	280	120	0	0	160	734	422	679	8	2.4
FA15	340	0	60	0	160	738	424	683	8	2.4
FA30	280	0	120	0	160	734	422	679	8	2.4
LM15	340	0	0	60	160	741	426	686	8	2.4
LM30	280	0	0	120	160	740	425	684	8	2.4

The total binder content was fixed at 400 kg/m^3^ and the water-to-binder ratio was maintained at 0.40 for all mixtures.

With the adopted w/b ratio and appropriate superplasticizer dosage, all mixtures achieved slump flow values in the range of 660–750 mm, corresponding to the SF2 class according to PN-EN 206:2014^48^.

### Research methods

The fresh-state consistency and flow characteristics of SCC were evaluated in accordance with PN-EN 206:2014^48^. Flowability was quantified using the slump flow test (spread diameter, mm). Passing ability was assessed by the L-box test (H_2_/H_1_), and viscosity-related behavior was characterized using the V-funnel test (flow time, s).

Compressive strength was determined on 150 × 150 × 150 mm cubes following PN-EN 12390-3^49^ using a 2000 kN testing machine. Tests were performed after 28, 56, and 90 days of water curing.

The static modulus of elasticity was measured in accordance with PN-EN 12390-13^50^ on cylindrical specimens (Ø150 × 300 mm) after 28, 56, and 90 days of water curing at 20 ± 2 °C.

Freeze–thaw resistance was evaluated using the F150 procedure^51^. Concrete specimens (*n* = 12), with a minimum dimension of 100 mm, were cured under standard conditions for 28 days prior to testing and then divided into two series: a freeze–thaw series (6 specimens) and a reference series (6 specimens) not subjected to cyclic freezing. The test specimens were exposed to 150 freeze–thaw cycles, each consisting of freezing in air at − 18 ± 2 °C and thawing in water at + 18 ± 2 °C, with a cycle duration of not less than 6 h. After completion of the test, freeze–thaw resistance was assessed by visual examination of surface damage, determination of mass loss, and measurement of compressive strength loss relative to the reference series. Concrete was considered to meet the F150 requirement when no visible damage was observed, mass loss did not exceed 5%, and compressive strength loss was not greater than 20% compared with the reference specimens. The freeze–thaw strength loss obtained from this procedure was subsequently used in the correlation analysis with hardened air-void system parameters.

The air-void system in hardened concrete was characterized after 28 days of curing according to PN-EN 480 − 11^52^, including total air content (A), micro-air content (A_300_), and spacing factor (L). These parameters were used to quantify the distribution and effectiveness of entrained air voids relevant to freeze–thaw durability. To support mechanistic interpretation, relationships between freeze–thaw strength loss and air-void parameters (particularly the spacing factor L) were further examined using correlation and regression analysis.

Microstructural observations of selected mixtures were performed using SEM/EDS. In addition, a proof-of-concept quantitative assessment of SEM image texture was carried out at a fixed magnification to provide an objective microstructural signature supporting the comparative interpretation. Because the available images were acquired in secondary-electron mode on fractured/crushed material and the dataset size was limited, the quantitative analysis was restricted to robust grayscale texture descriptors (GLCM-based contrast, homogeneity, and entropy) rather than attempting to infer intrinsic capillary porosity. Because only two images per mixture were available at the selected magnification, the quantitative SEM analysis was treated as exploratory and was not intended to provide a statistically representative description of microstructural variability. The quantitative SEM descriptors were therefore used as a complementary, supportive layer alongside the air-void system characterization and macroscopic durability indicators.

## Results

### Fresh properties and workability

All SCC mixes achieved self-compacting performance within the targeted SF2/VS1 workability classes according to PN-EN 206:2014^48^. The measured slump flow, V-funnel time, and L-box ratio are summarized in Table 3. Overall, the results confirm that partial cement replacement with mineral admixtures can maintain satisfactory SCC workability within the adopted mix-design framework.

**Table 3:** Workability indices of the SCC mixture.

Mineral Admixture	Cement Replacement (%)	Slump Flow (mm)	V-Funnel (s)	L-Box Ratio (H_2_/H_1_)
None (reference)	0	670	8.0	0.92
GGBFS	15	660	9.0	0.90
GGBFS	30	650	9.5	0.88
FA	15	720	8.3	0.95
FA	30	740	8.0	0.96
LM	15	700	8.6	0.89
LM	30	720	8.4	0.91

Among the investigated SCMs, FA showed the most favorable effect on flowability, GGBFS slightly reduced flowability and increased viscosity, whereas LM provided balanced fresh-state performance. In all cases, the L-box ratios confirmed adequate passing ability and low blocking tendency, indicating that the applied proportions remained suitable for SCC production. These trends are consistent with the known effects of FA particle morphology, slag-related water demand, and the filler action of limestone powder^14,16,17,19,20^.

### Compressive strength development

**Fig. 1:**
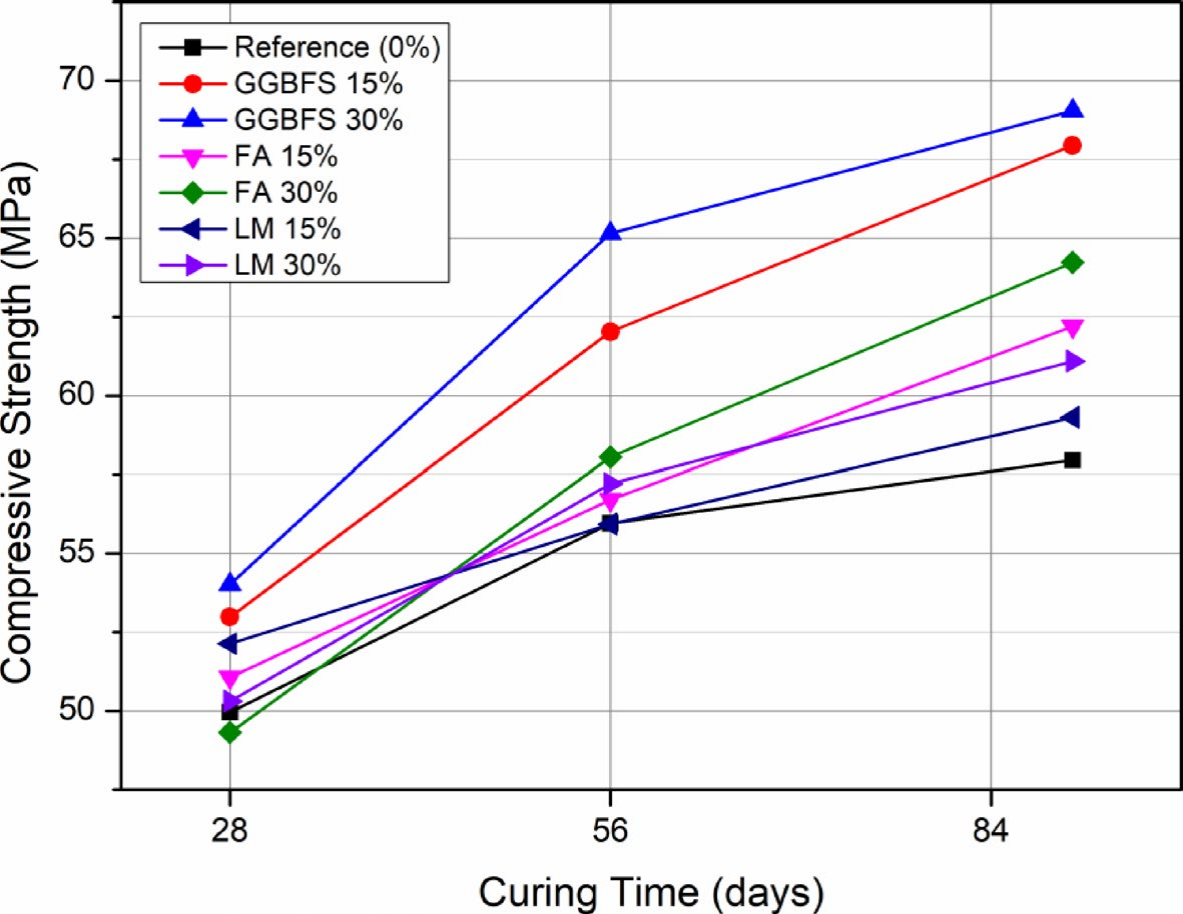
Development of compressive strength of SCC mixes with various mineral admixtures over curing periods of 28, 56, and 90 days. Plotted values represent mixture means; exact values and standard deviations are reported in Table 4 (*n* = 6 per mixture and curing age).

The compressive strength development of the SCC mixtures at 28, 56, and 90 days is presented in Fig. 1; Table 4. Numerical values and their variability are reported in the corresponding tables as mean ± standard deviation. All mixtures exhibited progressive strength gain with curing time, indicating ongoing hydration and, in the case of reactive SCM systems, continued secondary reactions. Overall, GGBFS provided the most pronounced late-age strength enhancement, FA showed a delayed but positive contribution, and LM produced more moderate gains.

**Table 4:** Compressive strength (MPa).

Days	Reference (0%)	GGBFS 15%	GGBFS 30%	FA 15%	FA 30%	LM 15%	LM 30%
28	50 ± 1.0	53 ± 1.5	54 ± 1.2	51 ± 1.0	49 ± 1.1	52 ± 1.3	50 ± 1.4
56	56 ± 1.1	62 ± 1.2	65 ± 1.5	57 ± 1.4	58 ± 1.5	56 ± 1.4	57 ± 1.3
90	58 ± 1.5	68 ± 1.7	69 ± 1.1	62 ± 1.2	64 ± 1.2	59 ± 1.7	61 ± 1.2

The strength ranking became more distinct with curing time. GGBFS-modified mixtures showed the strongest later-age response, which may be attributed to continued slag hydration and additional hydrate formation, leading to progressive matrix refinement^10,28^. FA mixtures also exhibited a beneficial later-age trend, consistent with slower but sustained pozzolanic activity and gradual densification of the binder matrix^16,17^. In contrast, LM primarily acted through a filler-driven mechanism, improving packing and matrix uniformity rather than contributing substantially through chemical reactivity^19,20^.

One-way ANOVA confirmed statistically significant differences in compressive strength among mixtures at 90 days (F = 55.76, *p* < 0.000001), supporting the observed ranking in strength development. The two-way ANOVA results (Table 5) further indicate significant effects of curing age, mixture type, and their interaction, confirming that SCM type influenced not only the absolute strength level but also the rate of strength development.

**Table 5:** Two-way ANOVA for compressive strength f_c_. Model: f_c_ ∼ Age + Mixture + Age × Mixture, Dataset: 126 observations (3 curing ages × 7 mixtures × 6 replicates).

Source of variation	df (num, den)	F	*p*-value	Partial η^2^
Age (28, 56, 90 days)	2, 105	837.15	2.98 × 10^−65^	0.941
Mixture (7 levels)	6, 105	90.17	3.86 × 10^−39^	0.837
Age × Mixture	12, 105	11.22	4.08 × 10^−14^	0.562

Partial η^2^ is reported as an effect size measure. Significant main effects of Age and Mixture and a significant Age × Mixture interaction indicate that SCM type modifies the rate of strength development. Post-hoc pairwise comparisons were performed using Tukey HSD (α = 0.05), as summarized in Table 6.

Tukey HSD post-hoc comparisons (Table 6), based on the compressive strength results summarized in Table 4, show that the mixture ranking evolves with curing time. At 28 days, GGBFS15, GGBFS30, and LM15 formed the highest-strength group (a), whereas the remaining mixtures (including the reference and both FA mixes) were not significantly different and belonged to a lower group (b). At 56 days, GGBFS30 achieved the highest compressive strength and formed a distinct group (a), followed by GGBFS15 (b), while all remaining mixtures clustered in a single lower group (c). At 90 days, both GGBFS mixtures constituted the top group (a) with no significant difference between 15% and 30% replacement levels; FA mixtures formed an intermediate group (b); LM mixtures showed a moderate improvement (c); and the reference mix exhibited the lowest strength as a distinct group (d) (Table 6).

**Table 6:** Tukey HSD post-hoc grouping for compressive strength at 28, 56, and 90 days (mean ± SD, MPa). Values sharing the same letter within a given age are not significantly different (α = 0.05).

Age (days)	Reference (0%)	GGBFS 15%	GGBFS 30%	FA 15%	FA 30%	LM 15%	LM 30%
28	50.0 ± 1.0 (b)	53.0 ± 1.5 (a)	54.0 ± 1.2 (a)	51.1 ± 1.0 (b)	49.3 ± 1.1 (b)	52.1 ± 1.3 (a)	50.3 ± 1.4 (b)
56	56.0 ± 1.1 (c)	62.0 ± 1.2 (b)	65.2 ± 1.5 (a)	56.7 ± 1.4 (c)	58.1 ± 1.5 (c)	55.9 ± 1.4 (c)	57.2 ± 1.3 (c)
90	58.0 ± 1.5 (d)	68.0 ± 1.7 (a)	69.1 ± 1.1 (a)	62.2 ± 1.2 (b)	64.2 ± 1.2 (b)	59.3 ± 1.7 (c)	61.1 ± 1.2 (c)

Different letters in parentheses indicate statistically significant differences between mixtures at the same curing age (Tukey HSD, α = 0.05).

### Modulus of elasticity

**Fig. 2:**
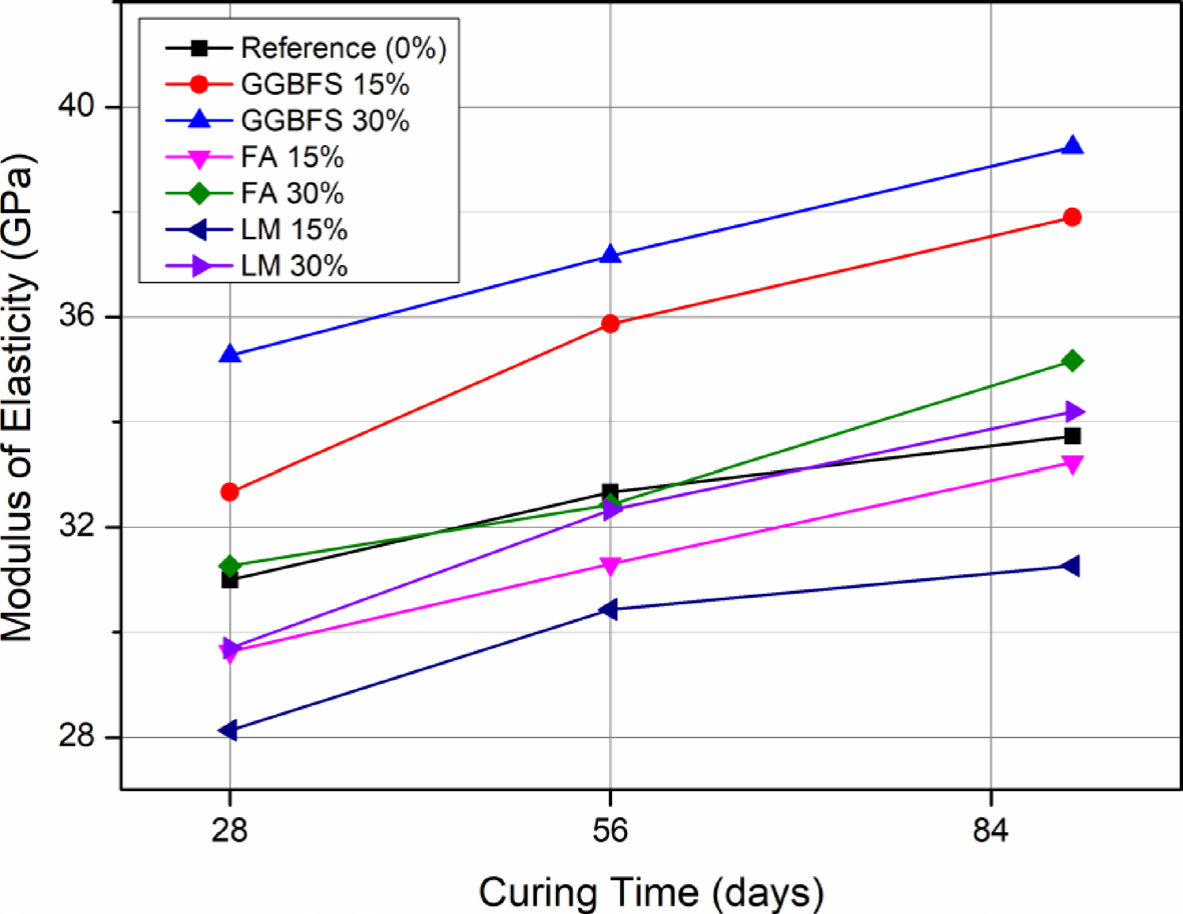
Development of modulus of elasticity of SCC mixes with various mineral admixtures over curing periods of 28, 56, and 90 days. Plotted values represent mixture means; exact values and standard deviations are reported in Table 7 (*n* = 3 per mixture and curing age).

The modulus of elasticity of the SCC mixtures increased with curing time, as shown in Table 7; Fig. 2, reflecting the continuing development and densification of the cementitious matrix^14^. The extent of stiffness development depended on SCM type and replacement level, and broadly followed the same hierarchy as compressive strength.

**Table 7:** Modulus of elasticity (GPa).

Days	Reference (0%)	GGBFS 15%	GGBFS 30%	FA 15%	FA 30%	LM 15%	LM 30%
28	31 ± 0.8	33 ± 0.9	35 ± 1.0	30 ± 0.7	31 ± 0.7	28 ± 0.8	30 ± 0.8
56	33 ± 0.9	36 ± 1.0	37 ± 1.1	31 ± 0.8	32 ± 0.8	30 ± 0.9	32 ± 0.9
90	34 ± 1.0	38 ± 1.1	39 ± 1.2	33 ± 0.9	35 ± 0.9	31 ± 1.0	34 ± 0.9

GGBFS produced the strongest stiffness enhancement, consistent with its contribution to later-age matrix refinement and the close relationship between elastic modulus and compressive strength^28^. FA mixtures showed a more gradual increase in stiffness, in line with their delayed but sustained pozzolanic contribution^16,17^. LM had a more moderate effect, suggesting that filler-driven packing and improved paste continuity can support elastic performance even in the absence of strong chemical reactivity^19,20^.

Two-way ANOVA indicated significant main effects of curing age and mixture type on the modulus of elasticity (Table 8), whereas the Age × Mixture interaction was not significant (*p* = 0.526). This suggests that stiffness increased with time across all mixtures and that the relative ranking between mixtures remained broadly stable.

**Table 8:** Two-way ANOVA for modulus of elasticity E_c_. Model: E_c_ ∼ Age + Mixture + Age × Mixture, Dataset: 63 observations (3 curing ages × 7 mixtures × 3 replicates).

Source of variation	df (num, den)	F	*p*-value	Partial η^2^
Age (28, 56, 90 days)	2, 42	92.65	3.98 × 10^−16^	0.815
Mixture (7 levels)	6, 42	65.38	1.03 × 10^−19^	0.903
Age × Mixture	12, 42	0.93	0.526	0.210

Partial η^2^ is reported as an effect size measure. Post-hoc pairwise comparisons were performed using Tukey HSD (α = 0.05), as summarized in Table 9.

Tukey HSD post-hoc comparisons (Table 9) confirm that the GGBFS mixtures consistently formed the highest group at later ages, while the remaining mixtures exhibited intermediate or lower stiffness levels depending on age.

**Table 9:** Tukey HSD post-hoc grouping for modulus of elasticity E_c_ at 28, 56, and 90 days (mean ± SD, GPa). Values sharing the same letter within a given age are not significantly different (α = 0.05).

Age (days)	Reference (0%)	GGBFS 15%	GGBFS 30%	FA 15%	FA 30%	LM 15%	LM 30%
28	31.0 ± 0.8 (b)	32.7 ± 0.9 (b)	35.3 ± 1.0 (a)	29.6 ± 0.7 (c)	31.3 ± 0.7 (b)	28.1 ± 0.8 (c)	29.7 ± 0.8 (c)
56	32.7 ± 0.9 (b)	35.9 ± 1.0 (a)	37.2 ± 1.1 (a)	31.3 ± 0.8 (b)	32.4 ± 0.8 (b)	30.4 ± 0.9 (b)	32.3 ± 0.9 (b)
90	33.7 ± 1.0 (b)	37.9 ± 1.1 (a)	39.2 ± 1.2 (a)	33.2 ± 0.9 (b)	35.2 ± 0.9 (b)	31.3 ± 1.0 (c)	34.2 ± 0.9 (b)

Different letters in parentheses indicate statistically significant differences between mixtures at the same curing age (Tukey HSD, α = 0.05).

**Fig. 3:**
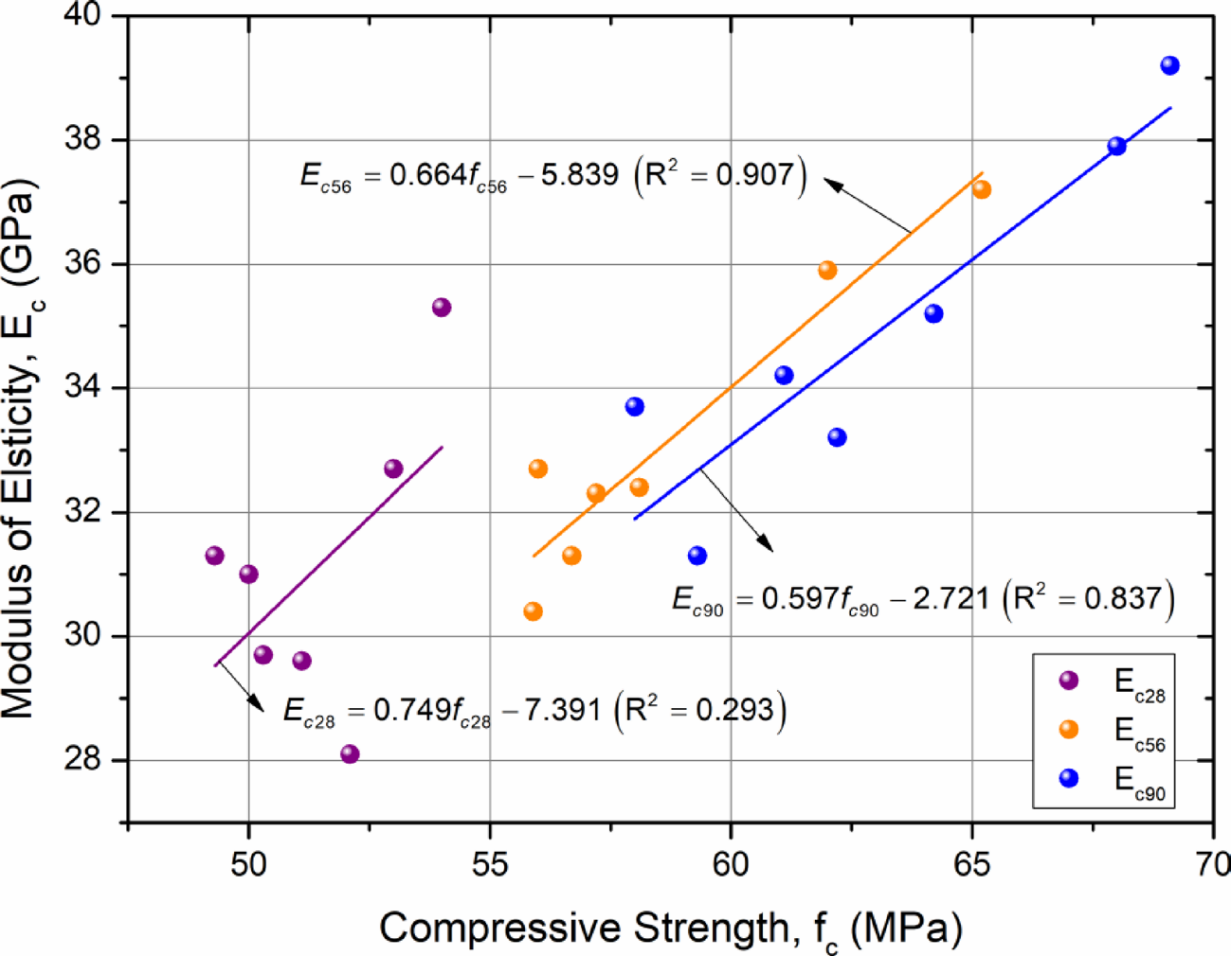
Age-dependent relationship between modulus of elasticity E_c_ and compressive strength f_c_ for SCC mixtures (28, 56 and 90 days).

To further support interpretation, the relationship between modulus of elasticity and compressive strength was examined at 28, 56 and 90 days (Fig. 3). The age-dependent E_c_–f_c_ plots show that the correlation becomes markedly stronger at 56–90 days, consistent with the statistically significant age effect and the relatively stable mixtures ranking. Accordingly, the fitted linear trends provide a simple empirical link between stiffness and strength for later-age SCC mixtures incorporating SCMs.

### Resistance to freeze–thaw

Freeze–thaw durability of SCC incorporating mineral admixtures was evaluated using the F150 procedure in accordance with PN-B-06250:1988^51^. For each mixture, specimens were conditioned in water for 28 days and then exposed to 150 freeze–thaw cycles. Mass change, visual condition, and residual compressive strength were determined after exposure, while companion specimens maintained under water curing served as references.

**Fig. 4:**
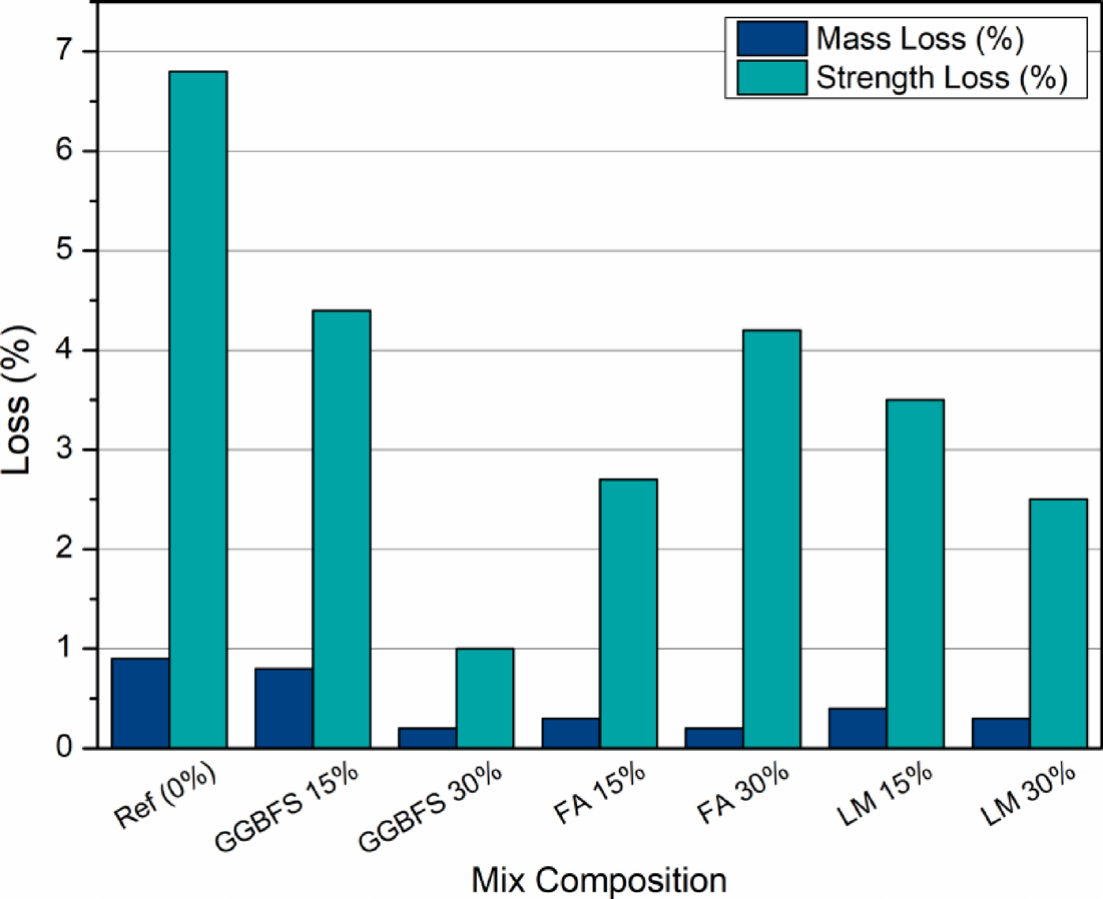
Freeze–thaw durability of SCC after 150 cycles: comparison of mass loss and compressive strength loss for all mixes tested. Plotted values represent mean results obtained from the F150 test; exact numerical values are given in Table 10 (freeze–thaw series: *n* = 6 specimens per mixture; reference series for strength comparison: *n* = 6 specimens per mixture).

The results are summarized in Table 10; Fig. 4. All mixtures satisfied the standard durability criteria, showing no visible cracking and remaining well below the acceptance thresholds for both mass loss and strength loss. Nevertheless, clear differences in performance were observed between binder systems. Overall, GGBFS provided the most favorable freeze–thaw response, FA also improved durability relative to the reference, and LM produced a moderate but consistent benefit.

**Table 10:** Freeze–thaw resistance (F150 method).

Mineral Admixture	Cement Replacement (%)	Mass Loss (%)	Strength Loss (%)	Cracking
None (reference)	0	0.9	6.8	none
GGBFS	15	0.8	4.4	none
GGBFS	30	0.2	1.0	none
FA	15	0.3	2.7	none
FA	30	0.2	4.2	none
LM	15	0.4	3.5	none
LM	30	0.3	2.5	none

The superior response of GGBFS-modified SCC suggests that slag contributed to a more durable matrix under cyclic freezing and thawing, likely through progressive refinement of the pore structure and reduced permeability. Fly ash mixtures also demonstrated improved freeze–thaw behavior relative to the reference SCC, but the two replacement levels did not follow a strictly monotonic trend. Although both FA mixtures showed very low mass loss, FA30 exhibited higher strength loss than FA15, indicating that surface scaling alone does not fully reflect internal damage. A plausible explanation is that, at the higher replacement level, the beneficial effects of improved workability and later-age pore refinement were not accompanied by a corresponding improvement in void spacing efficiency. In the present study, FA30 showed increased A300 but no clear reduction in spacing factor L relative to the reference system, which suggests that a larger fraction of fine voids did not fully translate into lower internal freeze–thaw damage. This interpretation is consistent with previous reports indicating that fly ash may improve freeze–thaw behavior through gradual matrix densification and improved compaction. However, the final durability response still depends on the combined action of pore refinement and the effectiveness of the protective void system^16^. LM mixtures showed a stable intermediate behavior, consistent with the beneficial effect of fine filler particles on matrix homogeneity and resistance to moisture-related damage^19,20^.

The reference SCC showed the highest losses, while still fulfilling the F150 criteria. Taken together, the results confirm that all investigated SCM systems can produce frost-resistant SCC within the present design framework, with the clearest benefit obtained for GGBFS at higher replacement level.

### Air-void system characteristics

To complement the freeze–thaw assessment, the hardened air-void system was characterized according to PN-EN 480 − 11^52^. The air-void analysis was performed using optical microscopy and computer-aided image analysis (Nikon SMZ1270 with Navitar optics, Nikon Instruments, Melville, NY, USA). For each mixture, three representative specimens were prepared as 150 × 100 × 20 mm sections and processed by cutting, grinding, polishing, and contrasting to obtain flat cross-section suitable for image acquisition. This method provides quantitative descriptors of entrained-air distribution and efficiency, which directly govern frost resistance through pressure-relief capacity and saturation sensitivity during cyclic freezing and thawing.

**Fig. 5:**
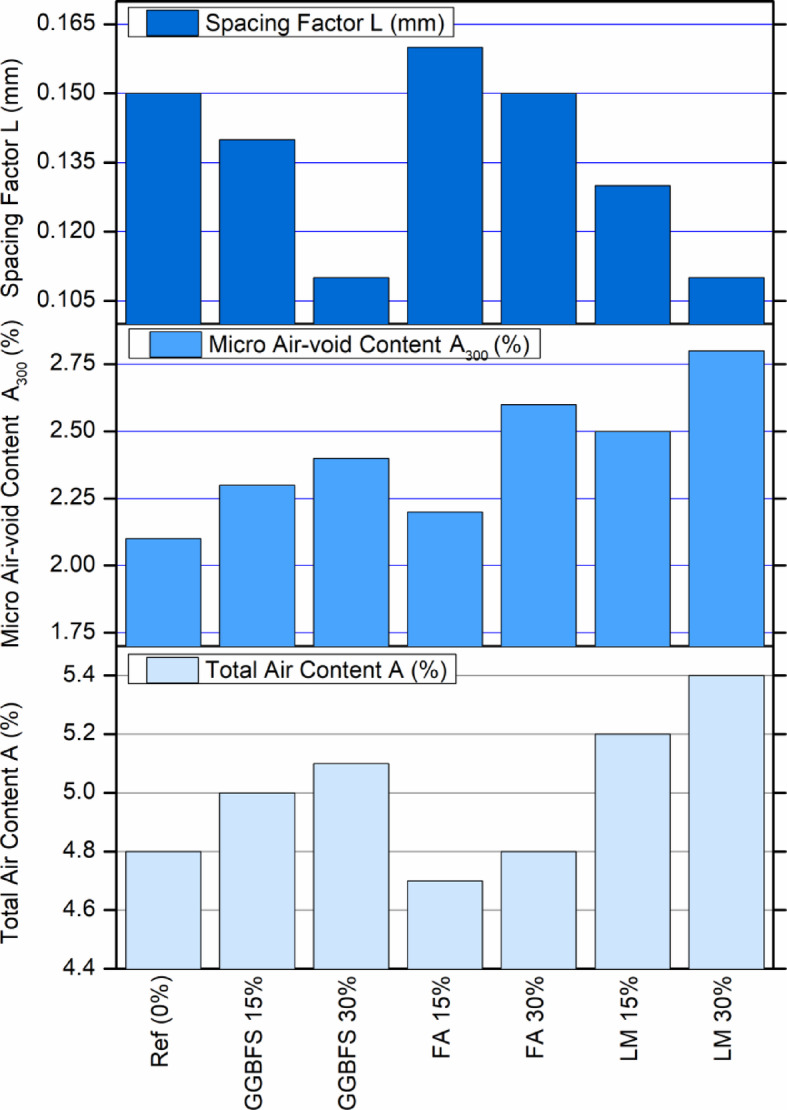
Air-void structure of SCC mixes: spacing factor L, micro air-void content A_300_, and total air content A. Plotted values represent mean results from image-based air-void analysis; exact numerical values are reported in Table 11 (*n* = 3 specimens per mixture).

The resulting air-void parameters are summarized in Table 11 and visualized in Fig. 5. Although total air content varied only slightly between mixtures, the spacing factor L and micro air-void content A_300_ provided clearer differentiation between SCM systems. In particular, the lowest spacing factor was observed for GGBFS30 and LM30, indicating a more closely spaced protective void network, while the highest A_300_ values were obtained for LM30 and FA30, suggesting an increased proportion of fine protective voids.

**Table 11:** Void structure – total air content A, spacing factor L, and micro air-void content A_300_.

Mineral Admixture	Cement Replacement (%)	Total Air Content, A [%]	Spacing Factor, L [mm]	Micro Air Void Content, A_300_ [%]
None (reference)	0	4.8	0.15	2.1
GGBFS	15	5.0	0.14	2.3
GGBFS	30	5.1	0.11	2.4
FA	15	4.7	0.16	2.2
FA	30	4.8	0.15	2.6
LM	15	5.2	0.13	2.5
LM	30	5.4	0.11	2.8

These results indicate that freeze–thaw performance cannot be interpreted on the basis of total air content alone. Instead, the efficiency of the protective void system appears to depend primarily on void spacing and the fine-void fraction. This is particularly evident when comparing mixtures with similar total air contents but different freeze–thaw responses, which suggests that the spatial distribution of voids is more important than their overall volume within the investigated range.

**Fig. 6:**
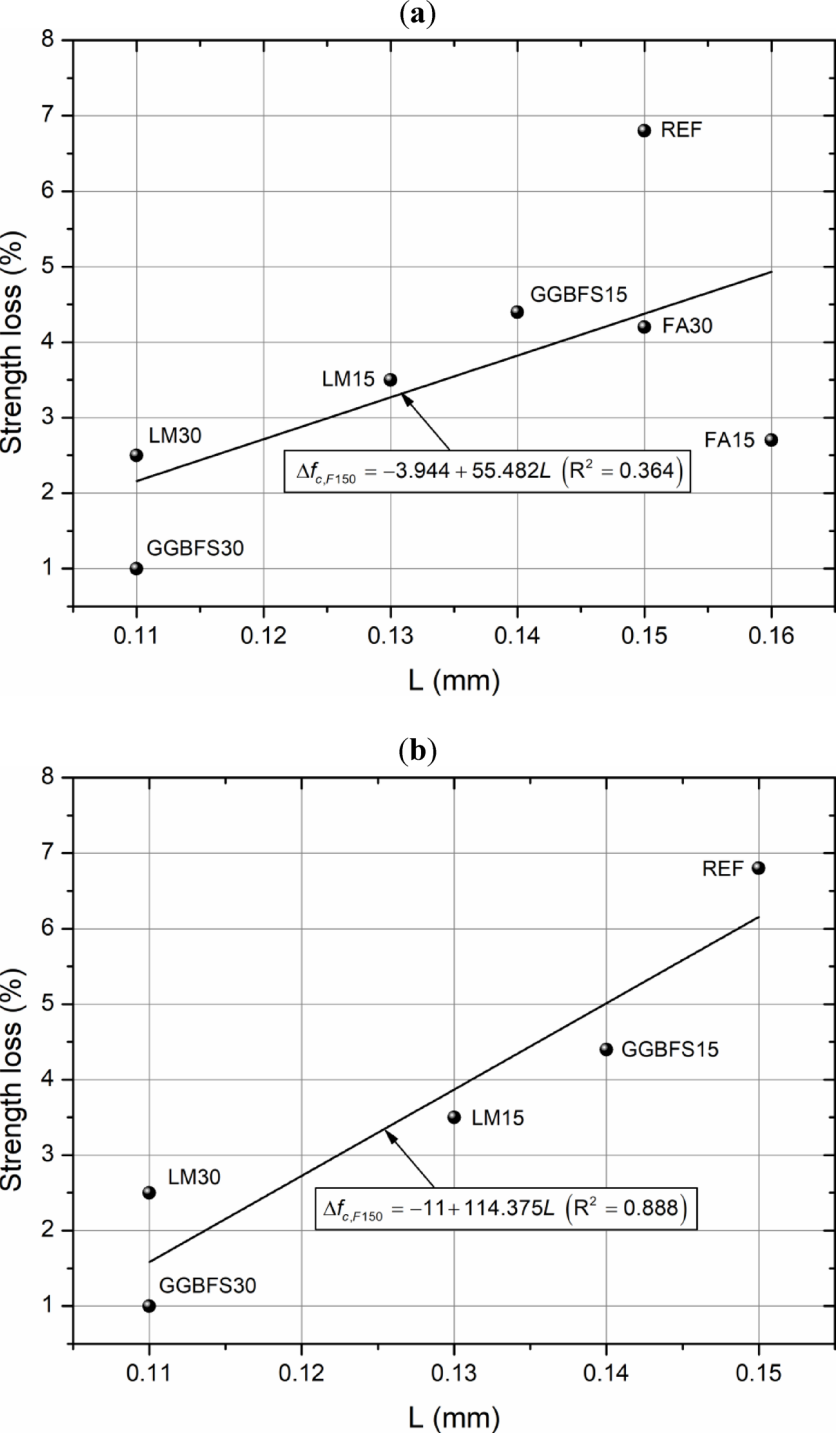
Freeze–thaw strength loss after F150 as a function of spacing factor *L*: (a) all SCC mixes; (b) reference + GGBFS + LM mixes (FA excluded). Solid lines denote linear regression fits.

To quantify this relationship, correlation and regression analyses were performed to relate freeze–thaw strength loss after F150 to the EN 480 − 11 air-void parameters, using the spacing factor L as the primary mechanistically motivated predictor within the investigated SCC systems. As shown in Fig. 6a, the global relationship between strength loss and L is only moderate when all mixes are considered together. This reflects SCM-specific differences, most notably the behavior of the FA-modified mixtures. In contrast, when FA mixes are excluded (Fig. 6b), a strong L-driven trend emerges for the reference, GGBFS, and LM systems, indicating that void spacing is the dominant practical predictor of strength loss in those mixtures. The deviation of FA15 and FA30 from this stronger trend suggests that, in FA-modified SCC, freeze–thaw performance is not governed by spacing factor alone. Rather, it appears to reflect a different balance between air-void characteristics and matrix development: FA mixtures showed an increase in A_300_, indicating a larger fraction of fine protective voids, but no comparable improvement in L. This helps explain why FA-modified SCC showed improved freeze–thaw resistance relative to the reference, yet did not follow the same L-controlled pattern as the GGBFS and LM systems. Accordingly, the present results support a tri-modal interpretation: GGBFS is predominantly L-driven, FA is more strongly associated with A_300_ and matrix-related effects, and LM benefits from the combined contribution of increased A/A_300_ and reduced L.

### Quantitative SEM/EDS image analysis

Quantitative SEM image analysis was conducted as a supporting assessment for three representative mixtures (reference, FA30, and GGBFS30). Because the available SEM dataset included multiple magnifications, all quantitative comparisons were restricted to a fixed magnification (4000×) to avoid scale-driven bias. For each mixture, two SEI micrographs acquired at 4000× were used for descriptor extraction (*n* = 2), while images acquired at other magnifications were retained for complementary qualitative illustration. Prior to analysis, the annotation band was removed, images were converted to grayscale, and intensity normalization was applied to minimize brightness/contrast differences.

Texture descriptors were computed using gray-level co-occurrence matrix (GLCM) metrics, including contrast, homogeneity, and entropy. As the images were acquired in secondary-electron mode on fractured/crushed material, the extracted descriptors are intended to quantify fracture-surface grayscale texture (i.e., topographic/contrast heterogeneity) rather than intrinsic capillary porosity. Accordingly, the quantitative SEM results are presented as a proof-of-concept complement to the macroscopic durability indicators and the air-void system characterization, rather than as a population-level statistical description. Given the limited number of images analyzed per mixture (*n* = 2), these texture descriptors should be interpreted as exploratory comparative indicators of fracture-surface grayscale heterogeneity at the analyzed scale, rather than as statistically representative microstructural parameters of the material.

**Fig. 7:**
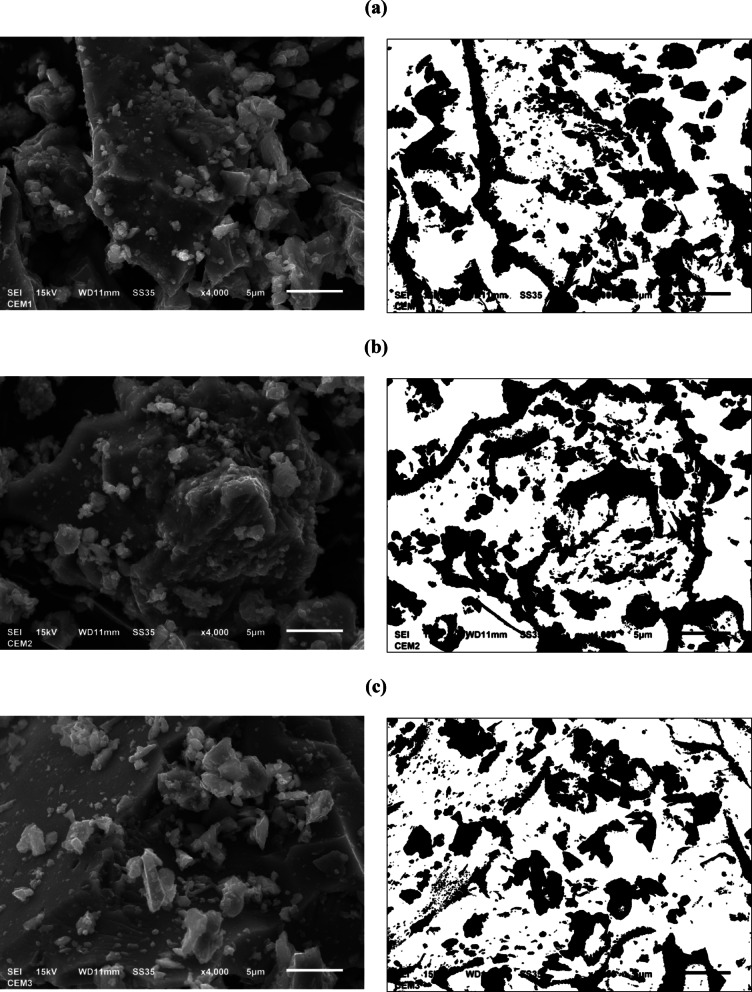
SEM micrographs of selected SCC mixtures at 4000× (SEI, 15 kV) with in-image scale bars and acquisition parameters, and the corresponding binary masks obtained by grayscale thresholding: (a) reference; (b) FA30; (c) GGBFS30. For quantitative image processing, the annotation band at the bottom of each micrograph was removed prior to grayscale normalization and descriptor extraction. Scale bar: 5 μm.

Representative SEM micrographs and the corresponding binary masks (used to illustrate the segmentation workflow) are shown in Fig. 7, while quantitative texture descriptors extracted from the fixed-magnification images are summarized in Table 12.

**Table 12:** Quantitative SEM texture descriptors (GLCM) obtained from SEI micrographs at 4000× for selected SCC mixtures. Values are reported as mean of two micrographs per mixture (*n* = 2), with the range in parentheses.

Mixture (code)	Contrast	Homogeneity	Entropy
Reference (CEM1, *n* = 2)	32.75 (29.83–35.67)	0.325 (0.291–0.358)	9.525 (9.157–9.893)
FA30 (CEM2, *n* = 2)	27.31 (24.97–29.66)	0.339 (0.289–0.390)	9.266 (8.724–9.809)
GGBFS30 (CEM3, *n* = 2)	34.56 (29.40–39.72)	0.249 (0.236–0.262)	9.969 (9.785–10.153)

The SEM-derived GLCM metrics (Table 12) provide a complementary quantitative signature differentiating the selected mixtures at the analyzed scale. FA30 exhibits lower entropy and higher homogeneity than the reference, indicating a more uniform grayscale texture of the fracture surface. In contrast, GGBFS30 shows higher entropy and lower homogeneity, which indicates a more heterogeneous grayscale texture at the analyzed scale. In the present context, this should not be interpreted as evidence of inferior matrix quality. Rather, it is consistent with a more complex and spatially variable fracture-surface texture associated with slag-modified hydration products and progressive matrix refinement. Because the descriptors were extracted from SEI images of fractured material, they reflect topographic and contrast heterogeneity of the fracture surface rather than intrinsic capillary porosity. Accordingly, the higher entropy of GGBFS30 is interpreted as a complementary microstructural signature of a denser but texturally more differentiated matrix, which remains consistent with its superior later-age mechanical performance and freeze–thaw resistance.

SEM–EDS analysis was performed on selected mixtures (reference, FA30 and GGBFS30) to provide representative microstructural evidence supporting the macroscopic performance trends. The EDS spectra were collected from local micro-areas within the representative SEM fields of view; therefore, the values reported in Table 13 should be interpreted as semi-quantitative local compositions of the analyzed regions rather than as bulk composition of the mixtures. The role of limestone powder was interpreted primarily based on the air-void system characteristics and freeze–thaw response, as its main contribution is expected to be physical (filler/packing) rather than strongly reactive. The elemental data reported in Table 13 correspond to local EDS acquisition regions selected within the representative SEM images in Fig. 7.

Compared with the reference mix, both SCM-modified mixtures exhibit a visibly more homogeneous fracture-surface appearance at the selected magnification (see Fig. 7). FA30 shows characteristic spherical ash particles embedded within the binder matrix; in some regions, partial reaction rims are visible, indicating ongoing secondary reactions at later ages. GGBFS30 displays a compact morphology of hydration products, consistent with progressive matrix refinement typical for slag-modified systems. At the same time, the quantitative GLCM results indicate that this compact matrix is associated with a more heterogeneous fracture-surface grayscale texture, which likely reflects the spatially variable arrangement of slag-related hydration products at the analyzed scale rather than increased internal damage. In contrast, the reference mixture shows a less compact fracture morphology, with locally visible unreacted grains and microcrack-like features, which is consistent with its comparatively lower durability indicators. These SEM observations are intended as qualitative support for the comparative interpretation rather than a quantitative porosity assessment.

**Table 13:** Elemental composition of selected SCC mixes after 90 days (SEM-EDS analysis).

Element	Reference (%)	FA30 (%)	GGBFS30 (%)
O	33.73	43.33	41.84
Mg	1.54	0.53	2.82
Al	8.63	3.25	3.38
Si	3.34	10.20	14.77
S	0.75	0.44	1.31
Ca	37.22	38.59	35.24
Mn/Fe	14.78	3.67	0.63

The reference mixture shows a Ca- and O-dominated signature (Ca 37.22%, O 33.73%), consistent with a hydrated Portland cement matrix. For FA30, the EDS results indicate increased silicon (Si 10.20%) relative to the reference (Si 3.34%), reflecting a more silica-enriched binder composition at the measured locations. GGBFS30 exhibits the highest silicon content (Si 14.77%) and a slightly lower calcium fraction (Ca 35.24%) compared with the reference, which is consistent with the presence of silica-rich hydrate assemblages typically associated with slag hydration and secondary reactions. While SEM–EDS is inherently local and semi-quantitative, the observed shift toward higher Si content in FA30 and especially GGBFS30 supports the macroscopic trends of improved later-age mechanical performance and freeze–thaw resistance for these mixtures.

Overall, the SEM–EDS observations provide microstructural context for the comparative results, indicating that reactive SCM systems (FA and particularly GGBFS) develop a distinct binder composition and surface morphology at 90 days, consistent with matrix refinement. These findings complement the durability interpretation based on the air-void system parameters and F150 response presented in the sections “Resistance to freeze–thaw”, “Air-void system characteristics”.

## Discussion and interpretation of results

The comparative results demonstrate that the type of mineral admixture controls not only strength development but also the dominant durability pathway in SCC, even when mixtures are produced within the same design framework. In particular, the present dataset enables a mechanistic differentiation of how GGBFS, FA, and LM influence frost durability through distinct microstructural and air-void signatures, which is rarely demonstrated in a single SCC matrix using a consistent testing protocol. This interpretation is aligned with sustainability-focused evidence that slag-based SCC can improve performance while reducing clinker demand and embodied energy^25,53,54^. In addition, a proof-of-concept quantitative SEM texture assessment (GLCM descriptors) for selected mixtures provides an objective microstructural signature supporting this differentiation (Table 12). Given the SEI fractured-surface imaging mode and the limited number of micrographs, these descriptors should be interpreted as texture/heterogeneity indicators rather than intrinsic porosity, yet they consistently distinguish the SCM-modified systems from the reference. When interpreted with appropriate caution, such morphological descriptors can still provide useful supportive insight into durability-related behavior by helping differentiate the fracture-surface texture associated with distinct microstructural development pathways.

The observed strength development is consistent with the different reaction mechanisms of the three SCMs. GGBFS produced the most favorable later-age response, which can be attributed to latent hydraulic reactivity and secondary reaction with portlandite, promoting additional C–S–H formation and progressive matrix refinement^10,28^. Fly ash showed the expected delayed but beneficial contribution, reflecting slower pozzolanic kinetics followed by gradual densification of the binder matrix at later ages^17,55^. Limestone powder followed a different pathway: its effect is primarily physical rather than reactive, operating through filler packing and nucleation effects that improve paste homogeneity and reduce capillary discontinuities^19,20,56^. Thus, although all three admixtures can support cement reduction, they do so through distinctly different mechanisms.

The elastic response followed the same hierarchy. The stronger effect of GGBFS on stiffness is consistent with the close link between modulus of elasticity, compressive strength, and matrix densification^14,28^. The more limited changes produced by FA and LM suggest that delayed pozzolanic contribution and microfiller-driven packing can preserve elastic performance, even if their effect on stiffness is less pronounced. Overall, the results indicate that the stiffness evolution is governed by the degree of microstructural refinement rather than by the nominal replacement level alone.

Freeze–thaw behavior should likewise be interpreted through the combined action of matrix refinement and the effectiveness of the protective air-void system. The superior frost resistance of GGBFS-modified SCC is consistent with a denser hydrate assemblage and reduced capillary connectivity, which limit water transport and internal damage accumulation during cyclic freezing and thawing^14,28^. Fly ash also improved freeze–thaw performance, but through a somewhat different route: its favorable particle morphology improves fresh-state compaction, while later-age pozzolanic action contributes to gradual pore refinement^16,57^. Limestone powder produced a more moderate durability benefit, best explained by physical densification and reduced water ingress rather than chemically driven transformations^19,20,58^.

This interpretation is consistent with previous studies on SCC exposed to freeze–thaw action. El-Mir and El-Zahab^59^ reported that the residual compressive strength of freeze–thaw-damaged SCC can be reliably assessed using UPV and that air-entrained SCC performed more favorably, especially at lower water-to-cement ratios. In turn, El Mir and Nehme^60^ showed that the effectiveness of air entrainment in SCC containing SCMs is governed not only by the presence of additional air voids, but also by the resulting balance between strength reduction and durability improvement. Together, these observations support the present view that freeze–thaw resistance of SCC depends on the interaction between matrix quality and the efficiency of the protective air-void system^59,60^.

A more discriminating explanation emerges when freeze–thaw response is related to air-void characteristics. In the present dataset, durability trends correspond more closely to spacing factor (L) and micro-air content (A_300_) than to total air content (A) alone. This suggests that frost durability is governed less by the absolute volume of entrained air and more by the efficiency of its spatial distribution within the matrix, which is consistent with established frost-resistance criteria^23^. Within this framework, GGBFS can be interpreted as primarily L-driven, FA as more strongly associated with A_300_, and LM as acting through combined stabilization of both air content and void spacing. This tri-modal interpretation provides a practical durability-oriented perspective for SCC mix optimization. The regression analysis linking freeze–thaw strength loss to spacing factor L (Fig. 6) further supports this interpretation. While the global relationship remains moderate when all mixtures are considered together, the clearer trend observed for the reference, GGBFS, and LM systems indicates that void spacing is the dominant practical predictor of strength loss in those mixtures. The deviation of FA mixtures suggests an additional durability mechanism not fully captured by L alone, consistent with their distinct air-void signature. A similar conclusion emerges from the literature on SCC with air-entraining admixtures and SCMs, where air-void characteristics were found to be essential for frost-related performance, but not sufficient on their own to explain the final mechanical response. In particular, the previously reported decrease in compressive strength associated with high air-entraining dosage confirms that durability enhancement must be interpreted together with changes in matrix structure and strength-bearing capacity^60^.

Regarding workability, the observed trends are consistent with the known effects of SCM particle morphology and fineness. Fly ash improved flowability through its spherical particle shape and lubricating effect, whereas slag tended to reduce flowability at higher replacement levels because of higher water demand and particle angularity. Limestone powder maintained stable SCC behavior through its filler action, which improves particle packing and paste volume. These observations confirm that SCC performance can be preserved across different SCM types when admixture dosage is properly optimized^61,62^. The present interpretation is also broadly consistent with earlier porosity-oriented studies on SCC, which showed that SCC may exhibit lower total porosity than normal concrete owing to its filler-rich and better-dispersed matrix, while still showing distinct absorption-related behavior because pore connectivity and transport characteristics are not governed by total porosity alone^63^.

In summary, the three SCMs improved SCC performance through distinct dominant mechanisms, which is the key comparative message of this study. GGBFS provided the strongest combined effect on late-age strength, stiffness, and freeze–thaw durability; FA primarily improved workability and delivered delayed strength gains; and LM contributed mainly through microfiller-driven densification and stabilization of the air-void network. From an applied standpoint, all three admixtures support meaningful cement reduction and thus contribute to more resource-efficient and lower-clinker SCC design^53,54,64^. Recent evidence also supports that eco-efficient SCC incorporating SCMs can maintain adequate workability while improving long-term performance when mixture proportions are properly optimized^65–67^.

Despite these benefits, broader implementation may still be influenced by practical constraints. Variability in SCM quality, particularly the chemical composition, fineness, and reactivity, may introduce performance scatter even at nominally identical replacement levels. In addition, long-term availability of high-quality FA and GGBFS may become more restricted in Europe due to industrial transitions. Future research should therefore extend this durability framework to alternative SCMs and develop predictive mix-design strategies that explicitly account for SCM variability and local availability. Overall, the combined air-void system analysis, regression-based durability linking, and complementary microstructural signatures provide a performance-oriented framework for SCM benchmarking in SCC.

Beyond the direct mechanical and durability effects discussed above, the use of SCMs has broader sustainability implications because partial clinker replacement directly reduces the environmental burden associated with cement production. Recent life-cycle assessments have shown that SCM-blended OPC concretes can achieve meaningful reductions in CO_2_ emissions, with reported decreases of about 15–29% depending on the SCM type, and even larger reductions when optimized blended systems are used. Likewise, material-level and mix-level LCA results indicate that the binder phase dominates concrete emissions, contributing roughly 71–95% of the total CO_2_ footprint, so reducing clinker content is one of the most effective strategies for lowering embodied carbon while maintaining structural-grade performance^68,69^. At the same time, previous research also shows that SCM-induced changes in microstructure can affect stiffness and longer-term mechanical response, with modulus of elasticity often being particularly sensitive to durability-related damage processes^70^. In this context, the present results support a dual interpretation of SCM use in SCC: clinker reduction is not only an environmental strategy, but also a performance-based design approach when elastic response, pore refinement, and durability are assessed together.

Despite the breadth of the present experimental program, several limitations should be acknowledged. First, the quantitative SEM texture analysis was based on a restricted image set and was therefore used only as an exploratory comparative tool rather than as a statistically representative microstructural characterization. Second, freeze–thaw durability was evaluated under a single exposure regime (F150), which provides a useful comparative benchmark but does not capture the full range of frost-related deterioration scenarios. Third, the durability assessment was focused on freeze–thaw response and air-void system efficiency, without extending to other relevant long-term deterioration mechanisms such as chloride ingress, carbonation, or coupled transport-related effects. Future research should therefore expand the durability framework toward multi-mechanism assessment and broader exposure conditions, while also incorporating larger microstructural datasets for more robust quantitative interpretation.

## Conclusions

This study provides a unified, head-to-head comparison of GGBFS, fly ash (FA), and limestone powder (LM) as partial cement replacements in self-compacting concrete (SCC), combining fresh-state performance, mechanical development, freeze–thaw response (F150), and air-void system characterization. The main conclusions are as follows:

1. GGBFS provided the strongest overall later-age benefit, delivering the highest strength and stiffness together with the best freeze–thaw resistance among the investigated SCM systems.
2. FA primarily improved fresh-state workability while maintaining satisfactory later-age mechanical performance and better freeze–thaw behavior than the reference mixture.
3. LM produced a moderate but consistent improvement, particularly by maintaining stable SCC workability and contributing to durability-relevant air-void refinement.
4. Freeze–thaw resistance was governed more by air-void efficiency than by total air content alone. In particular, spacing factor L and micro-air content A_300_ provided clearer differentiation of frost durability, while FA mixtures suggested an additional mechanism not fully captured by L alone.
5. SEM/EDS observations and exploratory quantitative SEM texture analysis provided complementary microstructural support for the comparative interpretation of the SCM effects.

Overall, the results confirm that up to 30% cement replacement with common SCMs can be achieved while maintaining SCC workability and meeting F150 durability criteria, with GGBFS providing the most favorable overall performance under the present design framework.

Future work should extend this framework to multicomponent binder systems, additional durability mechanisms (e.g., chloride ingress, carbonation, ASR-related effects), and more robust image-based microstructural quantification based on larger standardized datasets.

## Data Availability

The datasets generated and/or analysed during the current study are available from the corresponding author upon reasonable request.
